# Proliferation and regeneration of the healthy human urothelium: A multi-scale simulation approach with 16 hypotheses of cell differentiation

**DOI:** 10.1371/journal.pone.0325132

**Published:** 2025-06-20

**Authors:** Fabian Siegel, Angelo Torelli, Minca Mattis, Julian Debatin, Philipp Erben, Markus Gumbel

**Affiliations:** 1 Department of Biomedical Informatics, Medical Faculty Mannheim at the University of Heidelberg, Mannheim, Germany; 2 Competence Center for Algorithmic and Mathematical Methods in Biology, Biotechnology and Medicine, Mannheim University of Applied Sciences, Mannheim, Germany; 3 Clinic of Urology and Urosurgery, Medical Faculty Mannheim at the University of Heidelberg, Mannheim, Germany; University of Minnesota Medical School, UNITED STATES OF AMERICA

## Abstract

The urothelium as a stratified epithelium of the urinary tract is a slow regenerating tissue of different layers and shares similarities with the epidermis. Characteristics of its cell types, regeneration, tissue homeostasis, integrity and self organization are only partly understood. Precise computer-based models which investigate the cell kinetics of the urothelium from a theoretical perspective are beneficial for the regenerative medicine field and cancer research. Here an agent-based computer simulation based on the Glazier-Graner-Hogeweg (GGH) approach were developed and tested on 16 hypotheses of proliferation and differentiation mechanisms of the healthy urothelial tissue in steady state and tissue regeneration. A fitness-function was introduced for the quantitative comparison of respective models. The findings indicate that two similar hypotheses with the following features describe the healthy tissue best: 1) Progenitor cells either divide and differentiate in a stem cell fashion or proliferate according to the population asymmetry model in epidermis, 2) Basal cells divide symmetrically and differentiate into intermediate cells depending on contact to the basal membrane. 3) Intermediate cells do not proliferate but differentiate into umbrella cells when they are in contact with the medium.

## Introduction

### Motivation

The coordination of epithelial cell proliferation, differentiation and migration is crucial for tissue homeostasis and self organisation which is interrupted in case of tissue injury or during carcinogenesis. The urothelium is a stratified epithelium of the urinary tract and builds a barrier between blood and urine. It is a slow regenerating tissue with a turnover time of 3 to 6 months [1–3]. However, after injury there is an enormous regeneration capability of the uroepithelium within days after significant damage [1].

**Fig 1:**
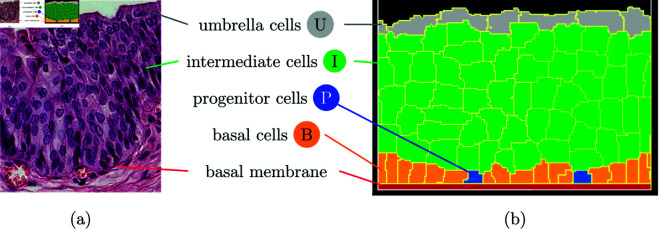
(a) Histological cut through the urothelium. (b) Simulation.

The urothelium structure shares similarities to the epidermis and is made of different layers. Both have one basal layer consisting of basal and progenitor cells occurring above of the basal membrane, which separates them from the deeper connective tissue. Above this are three to five layers of intermediate cells and ending with a single layer of specialized cells, which in the case of the urothelium are the umbrella cells. Fig 1 compares a histological cut and a simplified urothelium representation. Clinical and economically relevant diseases of the urinary tract of children and adults include malformations, infections and cancer [4,5].

The attributes of the urothelium are highly complex and the characteristics of each cell type of this tissue is not yet fully understood [6,7]. The development of precise theoretical and computational models for further understanding of urothelial regeneration and homeostasis will be beneficial for the regenerative medicine field and research on cancerogenesis of bladder cancer [6,8]. Cancerogenesis is even more complex and disruption of tissue hemoestasis is essential for cancer development [9].

The similarity of the anatomical structure between the epidermis and the urothelium suggests the development of new models for the urothelium which are influenced by models of the epidermis. For instance, Li *et al*. [10] discuss three hypotheses for cell proliferation in epidermis: 1) asymmetric division, 2) population asymmetry and 3) population asymmetry with stem cells (PAS). Therefore 16 different models for cell proliferation of urothelium that simulate these hypotheses were analyzed.

### Related work

Only few attempts to simulate the urothelium have been developed over the years to investigate and understand its proliferation. Carvalho *et al*. simulated the healthy urothelium by the means of a cellular-potts-model [11], which showed that stiffness or the adhesion to neighboring cells is ciritcal in the initial moments of bladder tumor development. There have been two papers by Eugene Kashdan [12], in which he and his colleagues simulate the urothelium as cellular automata, inspecting the influence of matrix metalloproteinase (MMP) and secreted inhibitors from the urothelial cells to demonstrate the development of invasive bladder cancer [12,13]. Similar work to ours has been done in other types of epithelium, such as the crypts of the small intestine [14] and of the epidermis [10]. The CompuCell3D-framework [15] used in our paper was used to explore the formation of cysts in autosomal dominant polycystic kidney disease by simulating the possible effect of the activation of the gene expression of cadherin-8 in cells in a renal tubule [16]. In this multi-scale model it was postulated that the decrease in contact inhibition and increase of proliferation could be the cause of the activation of cadherin-8, which was then confirmed by in vitro experiments.

The urothelium models presented in this paper are extensions of the models discussed in Torelli *et al*. [17] and they mimic the cell proliferation in great detail: Differential adhesion between cells is used as a mechanism for cell sorting. The voiding of the bladder is also considered, which is also essential for malignant transformation and solid tumor formation [18]. Additionally, contact-inhibition and apoptosis of cells is included. The focus of this paper is 2D simulation of the tissue.

## Material and methods

The findings in this paper are based on two steps: 1. 16 models of representing hypotheses of cell proliferation for the healthy tissue were defined and 2. simulated each model with at least 25 independent runs. The simulations were then quantitatively assessed with a fitness criteria based on cell order and volume of cells.

### Building blocks and biological processes

The healthy urothelium is a *flowing* tissue that is in a steady state: New cells are permanently born and older cells leave the tissue but the overall structure remains stable. Thus, our models contain birth- and death-processes which are either part of the lineage or caused by other factors.

It is generally assumed that there is enough nutrition within the tissue for the cell to thrive and proliferate if they do so according to their lineage. Nutrients diffuse from the lamina propria through the basal membrane and are consumed by adjacent cells [19]. When cells grow and have reached their maximum (target) size they undergo mitosis or differentiate. A cell can die, either through apoptosis, or mechanically through the process of voiding. The chance of apoptosis is set differently for each cell type and ranges between $0.1\;\cdot \;10^{-6}$ and $180\;\cdot \;10^{-6}$ per day. Voiding of the bladder occurs every six hours. Here, apical cells that are in contact with the bladder lumen are randomly removed. The chance for being washed out is about 2% per voiding. Even a progenitor cell can be washed away if it reaches the surface.

### Models

**Fig 2:**
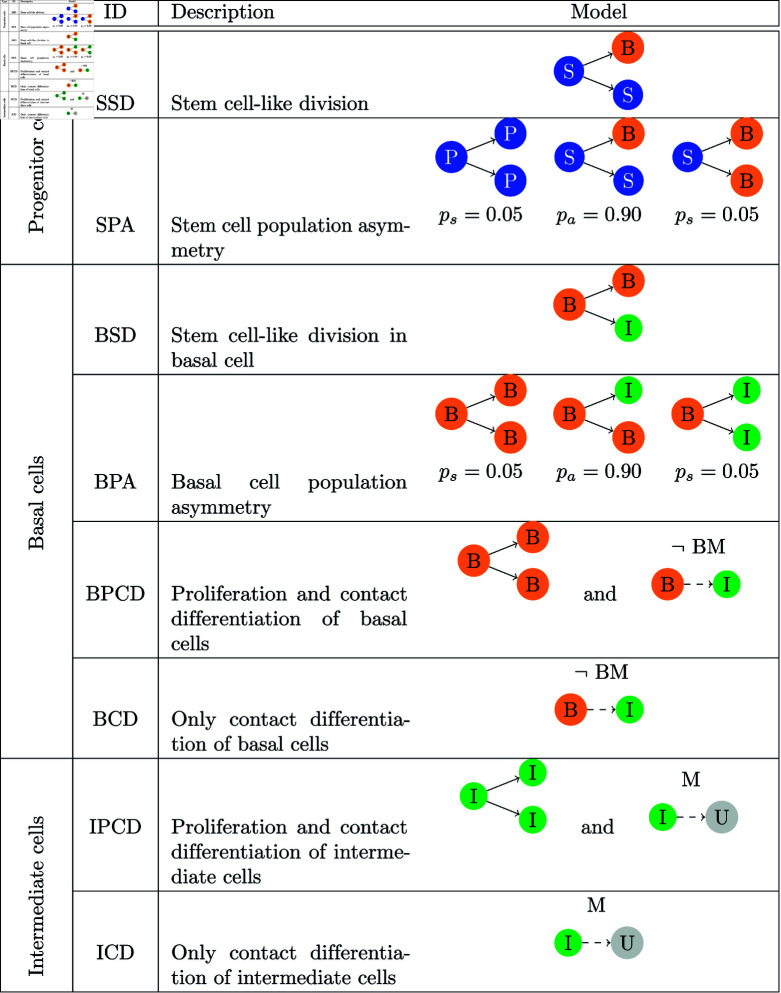
Possible proliferation and differentiation concepts for the three cell types progenitor (P), basal (B) and intermediate (I) cell (column Type). Column ID assigns a label for a specific proliferation or differentiation rule which refers to a specific model (column Model). Per cell type group one model can be chosen. In total we have 2·4·2=16 lineage models. Dividing cells are expressed by a plain line and transformation (differentiation) by a dashed line. Tranformation might happen either through contact (see ICD) or the loss of contact (see BCD).

Each model shares the properties defined above and additionally consists of a combination of the various proliferation and differentiation concepts for the three cell types progenitor, basal and intermediate cell as shown in Fig 2. These concepts are the building blocks that address biological and medical questions like: Are there symmetrical or asymmetrical cell divisions? Is cell differentiation contact driven? Do intermediate cells divide or not?

**Fig 3:**
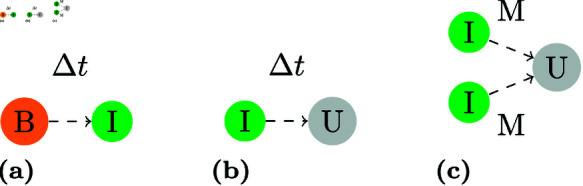
Possible proliferation and differentiation concepts that were excluded. A dashed line shows that a cell undergoes a direct transformation (differentiation) instead of a division, which might happen either after a certain amount of time like in a) and b) or through contact with the medium like in c). Latter one represents the special case of fusion, where two or more intermediate cells become one bigger umbrella cell to cover more surface area more quickly. This phenomenon very likely explains the findings of umbrella cells with multiple nuclei.

First of all, to address these questions we assume that cells cannot differentiate backwards and that the path is always P→B→I→U as suggested in Ho [7]. Additionally, we excluded time-dependent transformation (Fig 3a, 3b) and the special case of fusion (Fig 3c), which is discussed in [20], to concentrate on the building blocks with the highest potential for answering these questions. Fig 2 shows the 16 potential division and differentiation patterns chosen for a first analysis, classified by an ID, which is used later on to identify the models. Progenitor (oligopotent, stem cell like) and basal cells either divide and differentiate in a stem cell manner (SSD and BSD resp.) or according to the population asymmetry model in epidermis (SPA and BPA resp.). Two other hypotheses for differentiation of the basal cell are based on the differentiation into an intermediate cell when contact with the basal membrane is lost. The difference lies in the division: either with a symmetrical division into two basal cells or without any division at all (BPCD and BCD resp.). The last two hypothetical concepts were also tested for intermediate cells. The differentiation into an umbrella cell happens when the intermediate cell comes in contact with the medium (IPCD and ICD resp.).

### Tissue developments

The models introduced in section *Models* form 16 potential different cell lineages when combined. These cell lineages have been evaluated with computer simulations and each cell lineage model has at least *n* = 25 different runs, i. e. runs with a different seed value for the random number generators. Some of the simulations have several hundred simulation runs. Those models were of special interest and were tested thoroughly.

The initial condition for all simulations is a damaged urothelium where about 12% progenitor cells are randomly scattered on an otherwise empty basal membrane. This mimics the fast regeneration process of the tissue. 12% is an estimation of the number of progenitor cells in urothelium [19].

### Modelling techniques

The CompuCell3D framework [15] was utilized and extended with Python scripts. CompuCell3D is based on the Glazier-Graner-Hogeweg (GGH) approach [21] that is also known as the Cellular Potts Model (CPM). GGH models are Monte Carlo simulations with agent-based extensions. A biological cell is represented as a cohesive cluster of pixels (or voxels) placed on a two or three dimensional lattice. The time advances in discrete steps. Each step is called a Monte-Carlo-Step (MCS).

The core of any GGH model is the effective energy *E* that is minimized in the course of the simulation, reflecting the physical law to minimize the energy of a system. The composition of *E* describes the cell behaviors and interactions in a physical and biological manner – in our simulations *E* consists of the following components:

$$E=E_{V}+E_{S}+\lambda_{A}E_{A}$$ (1)

$E_{V}$ is a term that describes the energy for the cell’s volumes, *E*_*S*_ for the cell’s surface and *E*_*A*_ considers the adhesion of two cell’s surfaces. $E_{V}$ is the sum of the volume deviations as squared error for each cell (σ according to the notation in [15]). Each cell occupies a certain volume in the current state ($V_{a}(\sigma )$) and has a target volume ($V_{t}(\sigma )$), which is the volume a cell should reach to be healthy and varies depending on the cell type. Any deviation to the target volume increases the energy $E_{V}$ as follows: $E_{V}=\sum \left[\lambda_{V}(\sigma )(V_{a}(\sigma )-V_{t}(\sigma ){)}^{2}\right]$. The parameter $\lambda_{V}(\sigma )$ is cell type dependent and can be seen as an inverse compressibility factor, with lower values increasing fluctuations of a cell’s volume about its target volume. The energy *E*_*S*_ is implemented in similar fashion but instead of the volume, *E*_*S*_ takes the surface of each cell into account and weights the surface energy with $\lambda_{S}(\sigma )$. For the term *E*_*A*_ please refer to Swat *et al*. [15]. $\lambda_{V}$, $\lambda_{S}$ and $\lambda_{A}$ are weights that allows us to adjust the importance for each energy term. For instance, as cells are not compressible they must maintain their volume but can have flexible surface. This is achieved by setting $\lambda_{V}>\lambda_{S}$.

### Configuration

This section describes biological constants which could be determined and parameters, in particular those which are required for GGH simulations.

#### Space and time.

The space in which all the simulations run is 800 μm in width and 150 μm in height. We used a pixel density of 0.8 pixel / μm which leads to a lattice of 640×120×1 pixels. Note that the *z*-dimension is always 1 pixel to get 2D plane. The boundary conditions for the *x*-axis is set to periodical, while the *y*- and *z*-axes are fixed. Periodical in this case means that the left and right boundary are connected. The time resolution is 500 MCS per day and the urothelium was simulated for about two years (720 days) which is equivalent to a total of 360,000 MCS.

#### Cell properties.

**Fig 4:**
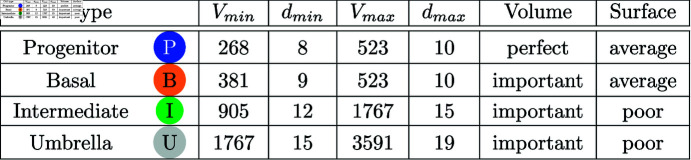
Cell properties [19]. Volumes *V* in $\mu {\text{m}}^{3}$, diameters *d* in μm. Columns *Volume* and *Surface* are adjusted with the weights $\lambda_{V}$ and $\lambda_{A}$, respectively.

**Fig 5:**
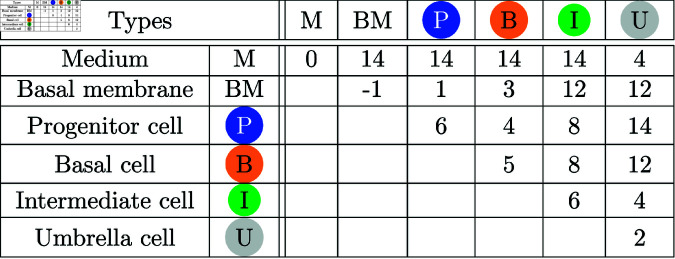
Surface tension values for the four cell types, the basal membrane (BM) and the medium (M). The values are according to CompuCell3D conventions: small values represent high adhesion, higher values less adhesion/greater repulsion.

Figs 4 and 5 list the remaining cell properties of the four cell types that were not already introduced as parameters. These are the minimum and maximum volumes and diameters as well as how rigorously a cell type should adhere to these volume and surface restrictions. The data was obtained by analyzing histological cuts [19,22–26]. We assume, by default, that cells have a circular or spherical shape. However, as cells are squeezed in a dense tissue, their shape is different. The more strict a cell type has to follow the surface restrictions, the stiffer a cell becomes. Stiffness limits the cell movement and it can be controlled by λ factor in Eq 1.

Contact inhibition is a well-known property of normal cells and contributes to the regulation of proper tissue growth, differentiation, and development. Contact inhibition of proliferation was included whereas contact inhibition of locomotion was not considered in this work. Former was postulated due to the fast healing times in contrast to the long turnover rates of the healthy tissue [1]. The cell proliferation rate in our simulations is accelerated by a factor of 50 when cells are not completely surrounded by other cells.

Finally, our models incorporate a cell sorting mechanism as the simulations by Torelli *et al*. [17] have shown that a correct layout of the cell layers can hardly be achieved without sorting. Cell sorting is achieved according to the differential adhesion hypothesis. It claims that cells move while minimizing the energy when cell membranes with specific adhesion molecules interact [27]. The combinations of all four cell types, the basal membrane and the medium results in 21 different surface tension values that are listed in Fig 5. The energies are set in a way that free floating cells would form an onion ring like structure with the basal membrane in the center, followed by progenitor and basal cells, then intermediate cells and on the surface umbrella cells. These adhesion energies were fixed in all simulations.

### Fitness function

In the following section we assume that we can measure the arrangement of cells and their volumes at specific points in time. Any simulation starts at time *t*_0_ = 0 and ends at time *t*_*e*_, typically *t*_*e*_ = 720 d. In between this range we can sample the tissue at time points $t_{0},t_{1},\ldots ,t_{e}$. For simplicity, in the following definitions *t*_*i*_ is omitted. We write *f* = *f*(*t*_*i*_).

#### Arrangement fitness function.

The arrangement fitness function *f*_*A*_ surveys to ensure that the cell strata are in the correct order. This is done by a function applying boolean error terms. It reaches an optimum of 1 if the urothelium reaches a state where the basal and progenitor cells layer is right above the basal membrane, followed by the various layers of intermediate cells and finally with one layer of umbrella cells, before the medium occupies the intraluminal space. In the worst case scenario, 0, the simulation does not create any cells. This equation is defined as followed:


$$f_{a}^{*}=\left\{ \begin{array}{cc} 1-\frac{E_{B}+E_{U}+E_{lib}+E_{opt}}{4} & \text{if number of layers }>0\\ 0 & \text{otherwise}. \end{array}\right.$$

The boolean error term *E*_*B*_ = 0 if the first layer is made of cell type basal or progenitor otherwise 1. Also *E*_*U*_ = 0 if the last layer is made of cell type umbrella otherwise 1. I.e. both values are 0 if the layout of basal and umbrella cells is perfect. *E*_*lib*_ (layers in between) is the percentage ($0\leq E_{lib}\leq 1$) of strata in between the first and last layer. It indicates the number of cells wandering away from their intended layer. Finally, *E*_*opt*_ = 0 if the number of cells in layer is between 3 and 7 or 1 otherwise.

The function $f_{A}^{*}$ is then calculated column-wise for every 25μm of the tissue resulting in a total of 31 samples. Out of these values, the average is taken and assigned to *f*_*A*_.

#### Volume fitness function.

Since no information was found in the literature search regarding the volume of each of the urothelial cell types, the relative volumes $v_{B,S}$,$v_{I}$,$v_{U}$ values were calculated as an average from a variety of histological pictures of the urothelium in a relaxed state (empty bladder) that were found in urology text books [19,22–26]. The results are $v_{B,S}=10\%$,$v_{I}=67\%$,$v_{U}=23\%$ of an averaged urothelium thickness of 85μm. With these values a non linear volume fitness function $f_{V}$ was created and is described as followed:


$$f_{V_{i}}=\frac{1}{4{\left(\frac{V_{S_{i}}-V_{I_{i}}}{V_{S_{i}}}\right)}^{2}+1}$$

$V_{S}$ and $V_{I}$ is the desired (should) and actual (is) volume of all cells of a specific type *i*. $V_{S}$ is equivalent to the width times the depth of the simulated urothelium times a height of 85μm. The overall fitness volume function $f_{V}$ is the arithmetic mean for all urothelium cell types (S,B), I, and U where S and B are considered as the group of cells laying on the basal membrane.

#### Total fitness.

The total fitness function for a specific point *t*_*i*_ averages the arrangement and volume fitness:


$$f(t_{i})=\frac{f_{v}(t_{i})+f_{a}(t_{i})}{2}$$

**Fig 6:**
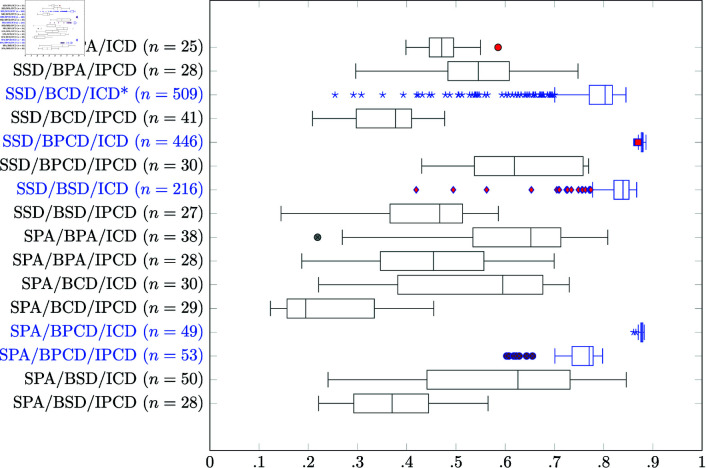
Fitness comparison of 16 different models. The five best models are depicted in blue. *n*: number of simulation runs. *: Contact model as published in [17].


The total fitness as applied in Fig 6 is the average total fitness value for all points in time:

$$f=\frac{1}{e+1}\sum_{i=0}^{e}f(t_{i})$$

with *e* + 1 indicating the number of time points.

### Software

The Python scripts for CompuCell3D are hosted as an open source project at Github (https://github.com/informatik-mannheim/Moduro-CC3D) with the name Moduro-CC3D. The simulation runs are analyzed with the Moduro toolbox that is also available at Github (see https://github.com/informatik-mannheim/Moduro-Toolbox).

## Results

### Tissue development

**Fig 7:**
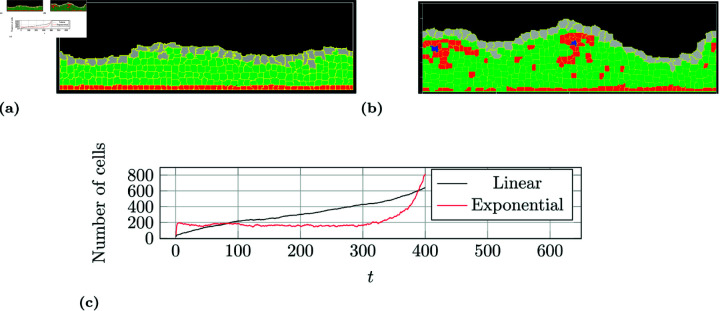
Examples of possible tissue development types. An example of a stable tissue development without progenitor cells taken from a SSD/BPA/ICD model is shown in a). An example of chaotic tissue development taken from a SPA/BCD/ICD model is shown in b). An example for overgrowing tissue development is not given since this would be a completely filled image without any medium (black background). Instead, the difference between linear and exponential overgrowth is shown in the form of a total cell count plot of the two. An example of atrophy tissue development is also not shown since it would be a completely black image (only medium).

All simulation runs of all the 16 models can be subdivided into one or more of the following tissue developments: stable, chaotic, linear and exponential overgrowing, and atrophy. A healthy tissue (Fig 1b) recovers fairly quickly (3 to 5 days) by creating a protective layer of umbrella cells and stays stable with a single layer of basal and progenitor cells on the basal membrane while three to five layers of intermediate cells lie in between. This is one of the two cases of stable tissue development. The other rare case is when the progenitor cells randomly wander to the surface and are washed away but thanks to the proliferation of the basal and or intermediate cells, enough cells are created to keep the tissue mass in balance (Fig 7a). A chaotic tissue development (Fig 7b) groups those simulations with a sequence of layers different from the one of a healthy tissue. This is often the result of the combination of a random detachment of the progenitor cell and lack of contact differentiation of basal cells (See BSD and BPA in Fig 2). When the balance between cell birth and cell death is disturbed in favor of proliferation, then the tissue overgrows. This is crucial in times of regeneration but unwanted and very dangerous in a healthy tissue, as it is the process seen in tumor development. Linear and exponential overgrowing tissue development types are shown in Fig 7c as plots of the total number of cells taken from two simulation runs with these characteristics (SPA/BCD/ICD for the linear and SSD/BPCD/IPCD for the exponential). The opposite of tissue overgrowth is referred to as tissue atrophy or decomposition.

Simulations can be categorized as a combination of these tissue development types. For example a chaotic tissue development can also be overgrowing or atrophy. An overview of all the types of tissue development for each model is listed in Table 1. These terms were introduced as another way to describe and group the results. A more detail description of a model with a healthy and a atrophy tissue development will be described in the next section.

**Table 1:** Occurrences of tissue development types in the different simulation runs of each model. Note that the columns can share a dependency. For instance, a tissue can develop chaotically and grow linearly.

Model	Stable	Chaotic	Linear growth	Exponential growth	Atrophy
SSD/BPA/ICD		•	•		
SSD/BPA/IPCD		•		•	•
SSD/BCD/ICD			•		•
SSD/BCD/IPCD				•	•
SSD/BPCD/ICD	•				
SSD/BPCD/IPCD	•			•	
SSD/BSD/ICD	•	•			
SSD/BSD/IPCD				•	•
SPA/BPA/ICD			•		•
SPA/BPA/IPCD			•		•
SPA/BCD/ICD			•		•
SPA/BCD/IPCD				•	•
SPA/BPCD/ICD	•				
SPA/BPCD/IPCD	•			•	
SPA/BSD/ICD			•		•
SPA/BSD/IPCD				•	•

### Fitness of the models

The computer simulations mentioned in section *Tissue developments* were compared according to a fitness criteria (see section *Fitness function*) calculated over the course of 720 simulated days. A fitness of 1 indicates a perfect healthy tissue whereas 0 represents a maximum disorder or a complete loss of the tissue. A summary of all the total fitness values of all the 16 models are shown in Fig 6 in form of a box and whisker plot.

Clearly, the lineage models SSD/BPCD/ICD and SPA/BPCD/ICD have the best fitness with a median fitness of 0.878 and 0.877 respectively. These two models also always develop into a healthy tissue which can be seen by the very narrow span of 0.018 and 0.021. On the contrary the span of the lineage models SSD/BCD/ICD and SSD/BSD/ICD are much broader with 0.591 and 0.448 respectively but still perform very well having a median fitness of 0.802 and 0.839 respectively.

**Fig 8:**
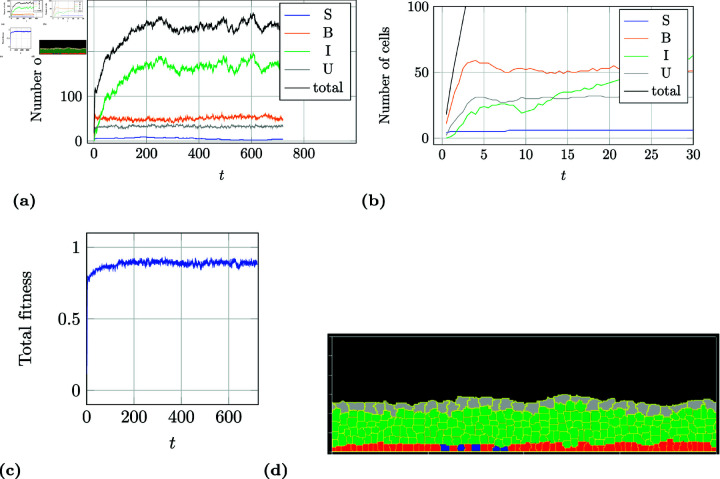
Example of a simulation run for a SPA/BPCD/ICD-model. These kind of models always create a stable, healthy tissues. The cell count from 0 to 720 days is shown in a) and from 0 to 20 days in b). After about 3 to 4 days the tissue is covered with umbrella cells (wound healing). The total fitness is shown in c) and an example for the simulated tissue at about 500 days is shown in d).

As an example, a simulation run with a healthy tissue development of a SPA/BPCD/ICD-model is described in more detail. Fig 8a shows the distribution of the number of cells over a time course of 720 days. At about 200 days a steady state has been reached. This observation is similar to all other simulations of this kind. Fig 8b shows the first 20 days. It can be seen that the tissue regenerates from just a few single progenitor cells in less than 5 days, which correlates to the findings from Lavelle *et al*. [28]. At this point in time the urothelium has formed its structure and is already covered with umbrella cells. Clearly, the number of intermediate cells still increases. Fig 8c shows the fitness curve for this simulation run. A fit of about 85 to 88 % is reached quickly and then the structure remains stable for the rest of the time. Fig 8d depicts a snapshot of the simulation at about 500 days. Here, a typical single layer of basal and progenitor cells is formed at the basal membrane followed by three to five layers of intermediate cells and finally a single layer of umbrella cells protecting all the layers beneath from the urine.

**Fig 9:**
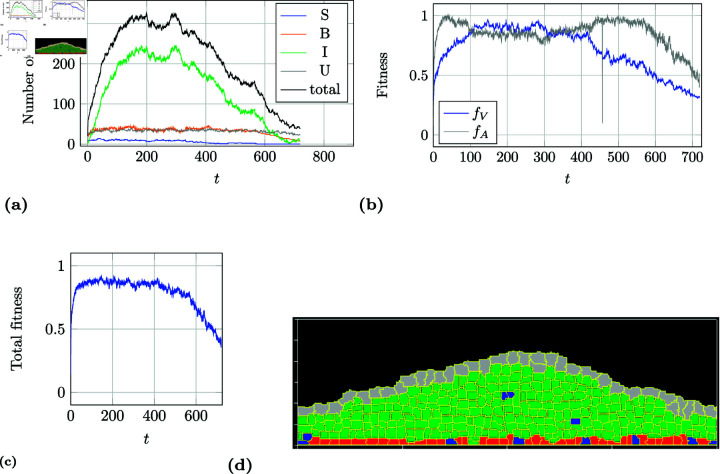
Example of a simulation run for a SPA/BSD/ICD-model. These models are intrinsically unstable. The cell count over the entire 720 days is shown in a). The volume ($f_{V}$) and arrangement fitness (*f*_*A*_) is shown in b) and the total fitness in c) (see section *Fitness function*). At day 573 no more progenitor cells are present in the tissue, which will result in total atrophy. An example for the simulated tissue at about 200 days is shown in d).

As a counter-example, a simulation run with an atrophy tissue development of a SPA/BSD/ICD-model was chosen. The total number of cells for each cell type over the course of 720 days can be seen in Fig 9a. By day five a protective layer of umbrella cells covers the entire surface of the basal membrane and all cells in between which results in an initial high arrangement fitness close to 1 (see Fig 9b). It continues to grow in a linear manner for 150 days growing stronger where more progenitor cells lie underneath forming a hill in the middle as can be seen in Fig 9c. This effect makes the arrangement fitness decline slightly because the number of intermediate cell layers should not be more than five or less then three. The tissue mass stays stable for approximately another 200 days maintaining 6 to 14 progenitor cells from day one. By being a model with progenitor cell population asymmetry the number of progenitor cells can vary greatly which only depends on pure chance. After the first year there is a decline in the number of progenitor cells which affects the entire tissue mass. This is also been reflected in both total number of cells (Fig 9a) and the volume fitness (Fig 9b) and therefore also the total fitness (Fig 9d). At day 573 the last progenitor cells divides symmetrically into two basal cells. Without a source of proliferation the tissue will continue to fade away until no cell would remain. This would have been the case if the simulation had continued past the 720 days.

### Adjusted parameters and derived measurements

**Fig 10:**
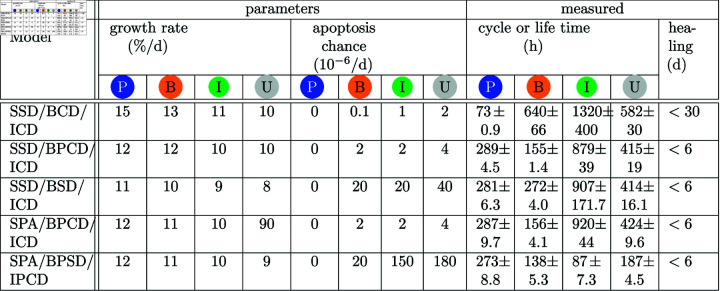
Parameters and derived data for the five best of the 16 models according the fitness function (see Fig 6). The left part shows the parameters for all cell types (S, B, I, and U) used to obtain a healthy tissue. The growth rate is in percent of the regular maximal cell volume $V_{max}$ (in $\mu {\text{m}}^{3}$) per day when cells are contact-inhibited. The apoptosis chance is the likelihood in 10^−6^per day that a cells undergoes apoptosis. The right part *measured* shows the derived data: cycle or life times is the average time that a cells of a specific cell type life before their undergo mitosis, differentiate or die. Healing shows the time until the urothelium is covered completely with umbrella cells when starting with an empty basal membrane and some progenitor cells (wound healing scenario).

Each model comes with a set of parameters which refine the building blocks and the general mechanisms. Here we describe those parameters that are unknown and adjusted to fit the models in the previous section. The remaining parameters are elaborated in section *Configuration*. Fig 10 shows the parameters which are varying from model to model. The building blocks SPA and BPA always have a likelihood of *p*_*s*_ = 0.05 for symmetrical and *p*_*a*_ = 0.9 for asymmetrical divisions. These numbers differ from those suggested in the epidermis model [10] where *p*_*s*_ = 0.1 and *p*_*a*_ = 0.8. If the chance for a symmetrical division in the urothelium was set to 0.1 the tissue very frequently lost all of its progenitor cells.

## Discussion

### Cell divisions in intermediate cells

The adult urothelium of the bladder is a stratified epithelium with three cell layers and cell types (basal-, intermediate-, and superficial or umbrella cells) similar to the skin epithelium. Other authors classify the urothelium shares similarities with a pseudostratified epithelium [29] similar to the respiratory tract, however, we decided based on current literature [30,31] to model it as an adult stratified epithelium, which closer models e.g. response to injury. The difference being that in pseudostratified epithelia, the Umbrella cells remain connected to the basal membrane. The traditional hypothesis of regeneration and differentiation suggests that a progenitor cell population is located in the basal cell layer and gives rise to all layers of urothelium and regenerates the mucosa in case of mucosal injury [7,32]. In contrast, other studies propose that intermediate cells can self-renew and generate superficial daughter cells, indicating an alternative pool of adult urothelial progenitors [6]. Also, it was shown that intermediate cells are highly proliferative and show rapid cell regeneration in response to injury or infection [33].

However, our findings clearly show an advantage of models with a small proportion of basal cells with progenitor cell division located at the basal membrane (SSD/BPCD/ICD and SPA/BPCD/ICD) in comparison to models with intermediate cell divisions.

Interestingly, the SSD/BCD/ICD model, where basal cells do not divide, is also able to create a healthy tissue. But this model fails when wound healing is considered as the regeneration time is about 30 days. The remaining four models all show a realistic wound healing time between 3 to 6 days as supported by the literature [34]. Three of the four remaining models in Fig 10 all have similar cycle times. Solely SPA/BPCD/IPCD also has proliferating intermediate cells but a slightly worse fitness (see 6). It is an open question whether this model could obtain a better fitness if its parameters would be better adjusted. Also, it is worth mentioning that according to the two best models it does not matter whether progenitor cells separate in a stem cell-like manner (SSD/PBCD/ICD) or according to the population asymmetry theory (SPA/PBCD/ICD).

Experimental data supports the finding that urothelial progenitor cells reside in the basal cell layer, because proliferation is observed in the lower cell layers. This was shown using label retention studies [35,36]. Further data by Gaisa *et al*. [37] sought to locate the stem cell niche in the human urothelium by demonstrating expansion from a single mutated stem cell by using naturally occurring mitochondrial DNA (mtDNA) mutations as a marker of clonal expansion. These data clearly showed based on histology that the human adult urothelium is maintained by progenitor cell-derived clonal fields of progeny.

### Adhesion to the basal membrane

Tissue homeostasis under “healthy conditions” needs coordination of cell proliferation, migration and differentiation which are interrupted during carcinogenesis. The main finding of this study is the high intrinsic stability of healthy tissue when a cell contact to the basal membrane and medium was supposed. The best two models (SSD/BPCD/ICD and SPA/BCPD/ICD) use contact differentation as a control mechanism. Even the SSD/BSD/ICD model that only uses differential adhesion for cell sorting can achieve an aceptable fitness. Which is in line with the model presented by Carvahlo and colleagues showing the importance of adhesion critically modulate the invasive of bladder cancer [11].

The normal urothelium expresses several adhesion molecules implicated in cell-cell and cell-matrix interactions, including E; P-cadherin, CD44 and several integrins [38,39]. These molecules are central for cell polarity and localization. Cadherins are transmembrane glycoproteins central for cell-cell and together with integrines for cell-matrix interactions [40]. It was shown that membranous P-cadherin expression was confined to the basal layer of normal urothelium [39]. Deregulation of these molecules, the “cadherine switch”, is clearly linked to tumor invasiveness in various epithelial malignancies and bladder cancer [41]. Integrins are cell adhesion receptors that bind to extracellular matrix ligands, cell-surface ligands or soluble ligands. They are important for cell attachment and control cell migration, cell cycle progression and programmed cell death [42,43]. Again alteration of these molecules are linked to tumor progression in bladder cancer [44].

The association of alterations in cell adhesion molecules with bladder cancer progression and invasion underline the importance of cell adhesion for normal patterns of urothelial tissue formation, regeneration, hemeostasis and self organisation. Our model implements qualitative cell behaviours to simulate the recovery after cell injury and cellular heterogeneity of the healthy urothelium. Further developments could be studying the application of oncotherapeutics in 3D [45]. This furthermore suggests further options using the proposed computational model identifying mechanisms by which an “altered cell population” may give rise to invasive tumors, which is essential to the understanding of the origin and biology of bladder cancer. Parts of the data our simulations relay on came from mouse models. Further steps are human-derived organoids, microphysiological systems and singel cell data can provide more valid information at the single-cell level for the urthelial homeostasis integrity and self organisation after injury.

## Conclusion

Our work evaluates, for the first time, several models of cell division and differentiation using a multi-scale approach with Compucell3D [15]. These simulations consider the complexity of cell characteristics and differentiation in regeneration of the adult urothelium after injury.
